# Best Practices in Fragile X Syndrome Treatment Development

**DOI:** 10.3390/brainsci8120224

**Published:** 2018-12-15

**Authors:** Craig A. Erickson, Walter E. Kaufmann, Dejan B. Budimirovic, Ave Lachiewicz, Barbara Haas-Givler, Robert M. Miller, Jayne Dixon Weber, Leonard Abbeduto, David Hessl, Randi J. Hagerman, Elizabeth Berry-Kravis

**Affiliations:** 1 Division of Child & Adolescent Psychiatry, Cincinnati Children’s Hospital Medical Center, Cincinnati, OH 45229, USA; 2 Department of Psychiatry and Behavioral Neuroscience, University of Cincinnati College of Medicine, Cincinnati, OH 45219, USA; 3 Department of Human Genetics, Emory University School of Medicine, Atlanta, GA 30322, USA; wekaufmann@ucdavis.edu; 4 MIND Institute, Department of Neurology, School of Medicine, University of California, Davis, CA 95817, USA; 5 Kennedy Krieger Institute, Johns Hopkins Medical Institutions, Baltimore, MD 21205, USA; budimirovic@kennedykrieger.org; 6 Behavioral Sciences-Child Psychiatry, Johns Hopkins University School of Medicine, Baltimore, MD 21287, USA; 7 Duke Department of Pediatrics, Duke University School of Medicine, Durham, NC 27710, USA; ave.lachiewicz@duke.edu; 8 Autism & Developmental Medicine Institute, Geisinger, Lewisburg, PA 17837, USA; bahaasgivler@geisinger.edu; 9 National Fragile X Foundation, McLean, VA 22102, USA; robby@fragilex.org (R.M.M.); jayne@fragilex.org (J.D.W.); 10 MIND Institute and Department of Psychiatry and Behavioral Sciences, School of Medicine, University of California, Davis, CA 95817, USA; ljabbeduto@ucdavis.edu (L.A.); drhessl@ucdavis.edu (D.H.); rjhagerman@ucdavis.edu (R.J.H.); 11 Departments of Pediatrics, Neurological Sciences, Biochemistry, Rush University Medical Center, Chicago, IL 60612, USA; Elizabeth_Berry-Kravis@rush.edu

**Keywords:** fragile X syndrome, clinical trials, treatment development, best practices

## Abstract

Preclinical studies using animal models of fragile X syndrome have yielded several agents that rescue a wide variety of phenotypes. However, translation of these treatments to humans with the disorder has not yet been successful, shedding light on a variety of limitations with both animal models and human trial design. As members of the Clinical Trials Committee of the National Fragile X Foundation, we have discussed a variety of recommendations at the level of preclinical development, transition from preclinical to human projects, family involvement, and multi-site trial planning. Our recommendations are made with the vision that effective new treatment will lie at the intersection of innovation, rigorous and reproducible research, and stakeholder involvement.

## 1. Introduction

Since the proposal of the “mGluR theory of fragile X syndrome (FXS)” over a decade ago, there has been an explosion of preclinical small molecule investigation in FXS leading to a number of mechanistically-targeted treatments moving into FXS human study. Despite significant pre-clinical data supporting translation of many drugs to humans, combined with some success in early-phase studies, no definitive large-scale placebo-controlled trials have met primary endpoints leading to drug use indications specific to FXS. As leaders and stakeholders in the field of FXS, we recognize the importance of issuing recommendations for methodology, including study design and strategies, which can maximize potential for success for the bench to bedside treatment development pathway. With this in mind, we review key aspects of the process of FXS treatment development with an eye towards ensuring that successful trials of new treatments, incorporating innovative research and stakeholder concerns, can be enacted.

## 2. Fragile X Clinical and Research Consortium Clinical Trials Committee

The National Fragile X Foundation (NFXF) has developed a Clinical Trials Committee (CTC) structure made up of Fragile X Clinical and Research Consortium (FXCRC) members, FXS clinicians, expert FXS trialists, outcome measure experts in the field, and family stakeholders to assist and support treatment developments. This committee reports and provides recommendations to the NFXF’s Facilitation of Research Oversight Group (FROG). The authors of this commentary all sit on the CTC. The CTC is designed to provide a one-stop point of contact to interface with industry, academic, and other partners seeking to move forward FXS-focused new treatment development at any stage. The CTC centralizes new treatment development support and advising in an orphan disease condition of great interest to potential business and academic partners. Moving forward the FXCRC Clinics have embraced the concept that, with many potential trials in FXS proposed, it will be critical to protect patients and optimize FXS participant resources through careful discussions about proposed trials and development programs with the CTC. The CTC will review preclinical data in early stages of drug development and subsequently help with recommendations regarding trial designs, outcome measures, and development strategy for agents showing promise on appropriate rigor preclinical and/or early phase clinical studies. 

Given the history of large-scale trial failures, and the large list of mechanistic targets and treatment development partners, it is critical for the FXS field to create a collaborative framework of expertise. The latter include those experts who led the first round of trials and representatives of affected families, who will collaborate in the design of trials and drug development paths that can capitalize on lessons learned and result in clear delineation of the drugs that have a positive impact in FXS and, ultimately, in the successful registration of these drugs for use by patients. Given this, it is advised that sponsors of all drug development in FXS that have reached the stage of multi-site Phase II or III trials, hold advisory meetings with and seek endorsement from the CTC, prior to use of FXCRC Clinics to carry out further development in FXS. Those seeking to engage the CTC may contact the National Fragile X Foundation CTC liaison (J.D.W.; jayne@fragilex.org) to initiate a dialogue. This recommendation is made to ensure that study sponsors receive accurate information facilitating the conduct of trials in FXS including broad stakeholder input. 

## 3. Preclinical Development

Experience with mouse models of FXS and other neurodevelopmental disorders has demonstrated their value as experimental systems for proof-of-principle assessments of new interventions [1]. Nonetheless, this work has also shown that phenotypes that are improved in mice do not necessarily translate directly to affected individuals [2]. Therefore, our field needs to emphasize the development of preclinical animal testing that can be evaluated in a similar manner in humans [3]. As important as the limited relevance of animal models to the human disorder, is the concern about reproducibility of animal data. The NIH has published guidelines promoting standardization of experimental paradigms and “best preclinical practices” for animal model work [4] and these have begun to be applied to neurodevelopmental disorders [5]. We recommend that any new treatment should be evaluated in blinded randomized experiments performed for multiple phenotypes in different domains (e.g., electrophysiological, molecular, behavioral), with some phenotypes being directly translatable to humans, and reversal of phenotypes shown in at least two independent laboratories [2] and ideally in several species, given the fact that rat and *Drosophila* models of FXS also exist. 

The presentation and publication of negative preclinical testing results is crucial to inform study design, outcome measure selection, and execution of first in human studies. Thus, this practice should be encouraged and is of importance to our field. The FXS field is challenged early on in drug development in part by a publication culture focused on “positive” preclinical data. Understanding which particular treatments may not improve murine or other animal phenotypes may be as important as understanding the aspects of success with a specific drug. 

In the same manner that human trials may not result in efficacious treatments for every individual with FXS, understanding variation within animal treatment response in vivo may provide early clues to features that may predict treatment response later in affected patients. Preclinical focus to date on murine findings has been on rescue or non-rescue with drug versus placebo treatment across various behavioral, molecular, and neuroanatomical assays and not on variation within treatment response. Further investigation into potential variability in preclinical treatment response may aide future planning when treatments move into human studies. 

Considering that mouse or other animal models work only provides evidence of treatment adequacy but not of specific outcomes, the possibility of skipping altogether in vivo model testing has been raised. Instead, in vitro evaluation of iPSC-derived neural cells offers the opportunity of testing directly cells from individuals with FXS and showing correction of cellular abnormalities, data that not only could be used for demonstrating treatment effectiveness but also subject selection [6]. However, this intriguing new approach does not test extension of cellular changes to neurobehavioral outcomes and will need to be systematically compared with current animal model standards prior to its full implementation [2]. In summary, the rigor, reproducibility, and specific findings of treatment development preclinical data should be evaluated in detail in planning for the scope and potentially specific aspects of the design of first in FXS human studies. 

## 4. Transition from Preclinical Studies to Human Subject Projects

Once preclinical work has demonstrated the scientific validity of a particular treatment through in vivo and/or in vitro studies, other elements of the experimental data can inform the subsequent steps. For initial human FXS trials, a project relying on single laboratory murine data or an iPSC model alone may be best served moving first into a human Phase Ib proof-of-concept target engagement study versus a brisk move into a large-scale first in FXS Phase II study. The latter would be more adequate for treatments supported by multiple preclinical studies providing convergent evidence and/or supported by small initial target engagement focused Phase Ib work in FXS. Drugs for which there is improvement of a directly translatable animal pharmacodynamic marker (e.g., event-related potential (ERP) abnormality) can be quickly tested initially in early phase human pharmacokinetic/pharmacodynamic (PK/PD) studies applying the biomarker prior to moving to larger clinical trials to help inform a large-scale study. Elements of adaptive trial design can be considered even in the earliest stages of human projects in FXS to facilitate the establishment of a predictive model incorporating biological markers and clinical outcomes. 

A process of de-risking large-scale multi-site projects in our field, utilizing well-conceived proof of concept early human studies, is quite possible. Such early work would need to include use of extensive objective and/or directly observable measures of brain function, communication, and behavior in order to determine potential clinically-relevant changes with treatment. Use of a crossover design, while potentially problematic in large-scale studies, given increased length, treatment expectation, and potential carryover effects on proxy outcome measures, may provide additional strength to Phase Ib projects that focus primarily on objective evaluations of target engagement. A rigorously designed Phase Ib trial in FXS may allow for an earlier go- or no-go decision on future larger scale multi-site studies of a drug, by requiring significantly less investment -in dollars and stakeholder commitment- than approaches with initial large-scale Phase II projects and by allowing for more informed biomarker and outcome measure selection based on a particular treatment or a particular cohort of individuals with FXS. 

Drugs targeting brain mechanisms in FXS with predominant effects on molecular, anatomical, and electrophysiological parameters, resulting in reversal of multiple and diverse phenotypes in animal models, would best move into human therapy through a Phase Ib PK/PD study looking at a broad range of objective outcomes, as optimal outcome measures are too difficult to predict in humans in this kind of scenario. Drugs that have an expected or known behavioral effect deemed important in FXS that has been replicated in multiple labs, and which target behavior as supportive care, may move faster to Phase II or Phase III trials with outcome measures directed at the predicted behavioral targets. Whether human studies entered in a gradual manner or with a rapid move into regulatory grade Phase II and III projects, the transition into and through the human stages of treatment development in FXS will benefit from many practice recommendations put forth below. Although early phase trials focused on safety or PK/PD could be conducted at a single site, studies evaluating efficacy benefit from a multi-site design. Regardless of size and scope, in the case of rare diseases like FXS, expertise in the disorder at the trial sites enhances the possibility of successful implementation and stakeholder satisfaction as discussed in more detail below.

## 5. Best Practices in Human Subjects Projects: Detecting Treatment Response

Heterogeneity in the clinical presentation of FXS relates to several factors including *FMR1* gene variations and background genetic and epigenetic effects, environmental stimulation, as well as negative life experiences. These factors lead to variability not only in the symptoms of FXS that individuals manifest but also in their response to treatment [2,7]. Likely, not all individuals with FXS will have a uniformly positive clinical response even to the best targeted treatments for FXS. Placebo effects are also remarkable in our field, especially when parent questionnaires regarding interfering behavior are used. Animal models have helped to guide us to specific treatments for patients, such as mGluR5 antagonists, the GABA-B receptor agonist arbaclofen, minocycline, metformin and other agents. However, trials with these agents have been potentially complicated by the presence of subgroups of responders, making entire cohort efficacy difficult to demonstrate. In this context, utilization of biomarkers such as EEG findings or responses in iPSC-derived neuronal cell cultures may be able to identify potential treatment responses for specific individuals with FXS, helping with stratification or selection of subjects for future trials.

The large placebo effect and selection of outcomes have been challenges in clinical trials in FXS. For example, in a well powered study reported by Berry-Kravis and colleagues (2016) there was a remarkable change in caregiver measures in the placebo group [8]. This significant placebo effect could have obscured true treatment-related changes in the active drug group. In the related field of autism spectrum disorder (ASD), a meta-analysis of 25 studies involving 1315 subjects, investigating placebo response in medication trials, demonstrated a “moderate effect size” for overall placebo response (Hedges’ *g* = 0.45, 95% confidence interval (0.34–0.56); *P* < 0.001) [9]. Reports like this raise the question whether we ought to expect a greater placebo effect when employing caregiver measures of abnormal behavior, in patients with neurodevelopmental disorders than in other populations [10]. If the answer is an affirmative one, that may reflect particularly high expectations among caregivers of individuals with developmental disabilities, including FXS, for treatments targeting key symptoms and reduction of disease burden [10], especially when widespread success of preclinical models is highly publicized. The strength of the placebo response in clinical trials for FXS supports the need to use more objective neurobehavioral and functional measures and/or to develop approaches that substantially mitigate biased reporting of treatment effects. Like all effective outcome measures, such “placebo-resistant” evaluations would need to be psychometrically sound (e.g., with known test-retest reliability), sensitive to change, and reflect improvements that are meaningful in the daily life of individuals with FXS. Ideally, the relationships between these measures and the underlying neural systems impaired in FXS should be understood so that in any given trial a measure can be chosen because it is proximal to the mechanism of action of the drug under study. It would also be useful if the clinical outcome measures have relevance to the neural cellular and circuit targets evaluated in preclinical studies [2].

Unfortunately, available outcome measures, including those in use in ongoing trials, generally do not meet all of the criteria outlined above [11]. Nonetheless, progress is being made in establishing the psychometric adequacy of several objective measures for quantifying change in important domains of general cognitive functioning [12], as well as specific areas of language [13] and executive function [14]. Preliminary results suggest that many of these promising measures are feasible for individuals of a wide range of ages and abilities, display minimal practice effects and strong test-retest reliability, and have good construct validity. At the same time, it is important to acknowledge that performance on these novel measures, as for most cognitive and behavioral measures, is likely to be affected by a number of skills and motivational factors and, thus, will reflect the functioning and complex interaction of multiple neural circuits that may result in attenuation of their sensitivity to detect drug efficacy. Also, we should acknowledge that placebo effects may occur even when using objective performance measures in individuals with intellectual disability [15]. 

Quantitative measures of pathophysiology are generally considered as potential continuous measures of efficacy; however, they can be equally considered as baseline predictors of treatment response. Genetic and blood-based molecular markers have already shown promise as identifiers of potential responders in drug trials in FXS [16]. While there is a high bar for official biological marker qualification as FDA-accepted surrogate biomarkers that can be used as regulatory endpoints in trials, use of biomarkers to identify target engagement, generally, and subgroups of potential responders with FXS is tractable in the near term. An approach like this may be able to parse the sometimes underappreciated heterogeneity of FXS in early trials, in order to guide subject selection or stratification in large-scale treatment trials. An example of this approach is ongoing work using single-dose placebo-controlled probe studies in adolescents and adults with FXS [17]. Another strategy, which should be incorporated as a standard procedure for all large-scale trials, is to collect blood samples for evaluation of genetic and other molecular markers. Development of brain- or blood-based markers will enrich research populations with responders to enhance treatment success and de-risk the perils faced by trials similar to unsuccessful past trials in FXS, which had broad inclusion criteria and relied on placebo-sensitive measures. For this reason, the NFXF has developed a NFXF Biobank^™^ program to receive biological samples and associated clinical data from persons with FXS including trial participants to provide a repository that will benefit biomarker understanding for the field regardless of final trial result.

Previous publications have examined the current status of outcome measures in FXS, providing practical recommendations and future directions [11,18]. Attributes considered critical for novel and improved outcome measures are the following: quantitative; reflecting brain circuits, function, or molecular pathways affected in FXS; relevant to experimental models of FXS; and reflecting quality of life of individuals with FXS. An example of these promising measures is event related potentials (ERPs), using repetitive auditory or visual stimuli and measuring habituation to the stimuli which is known to be abnormal because of inhibitory (GABA) deficits in FXS. Significant improvements in this measure were documented by Schneider and colleagues [19] in a controlled trial of minocycline compared to placebo and similar ERPs are being studied in the NeuroNext trial (FX-LEARN) with AFQ056 and parent-implemented language intervention (PILI, NCT02920892). Another type of promising measures are molecular markers reflecting core abnormalities in FXS, such as excessive protein production in FXS. For instance, levels of the matrix metalloproteinase 9 (MMP9) that are up-regulated in FXS have been shown to be reduced by administration of minocycline [20].

Biomarkers sensitive to change and correlated or predictive of clinical behavioral changes are of particular interest. Thus, assessment of how quantitative measures such as auditory ERPs, eye tracking and molecular biomarkers correlate with the clinical outcomes measured by behavioral ratings, cognitive and language tests, is critical. A number of recent phase 1b and 2a trials in FXS have incorporated all of these measures (NeuroNext AFQ056 (NCT02920892), metformin (NCT03479476), AZD7325 (NCT03140813), BPN14770 (NCT03569631)), such that the outcomes of these studies will improve our understanding of the relationships between biomarkers and clinical behavioral measures. An NIH multisite initiative called the Autism Biomarkers Consortium for Clinical Trials (www.asdbiomarkers.org) aims to identify a useful set of such tools.

Lessons learned from prior unsuccessful clinical trials in FXS suggest that greater emphasis on clinician- than caregiver-based measures; training systems for caregivers, clinicians, and other raters (e.g., teachers); evaluations in multiple settings (e.g., school, home, workplace); and behavioral ratings in real time on an electronic device or by videotaping, are approaches that could complement or replace current instruments, in particular behavioral rating scales. Finally, careful attention to other study aspects such as randomization and placebo inclusion in most early- and all late-phase trials; longer trial duration (to capture cognitive and adaptive changes); approaches to minimize placebo effects (e.g., placebo run-ins, enrichment in non-placebo responders, rater training); and younger cohort age would maximize the possibility of success.

## 6. Family Involvement

It is important that a family stakeholder voice is heard in FXS treatment development programs. Stakeholders can optimize recruitment and retention for FXS clinical trials. To accomplish this, it is important to provide parents with the most detailed possible information about all aspects of the trial. This would include: inclusion/exclusion criteria, length of the study, location, number/length/flexibility of visits, details on what will occur at each visit, and whether there is any travel/participation reimbursement available. It is imperative that family stakeholders understand the clinical manifestation targeted by the drug or intervention under study. It should also be noted that inclusion of an open-label extension phase following a placebo-controlled treatment will inherently boost study recruitment and retention. Enhanced communication with families about trial details, such as inclusion criteria and endpoints, should include the importance of adhering to the study design and avoiding attempts to “boost” reporting of a certain feature or behavior to facilitate study participation. Education on the implications of inaccurate reporting or embellishment of symptoms on the likelihood of obtaining appropriate results, which may benefit all stakeholders, is another key issue that should be communicated to families. 

Clinicians working with FXS family stakeholders often note the high level of motivation and commitment regarding treatments or clinical trials that may benefit their loved one with FXS. Given this zeal, parents/caregivers may be inadvertently drawn to a new treatment that may not be suitable for their family member with FXS. There are many reasons why a trial could not be an appropriate fit for a specific individual with FXS, including safety and efficacy concerns, inclusion or exclusion criteria, outcome measures, length of project and/or number of appointments required. From a caregiver viewpoint, it is important that drug trials have a protocol that includes an operations manual detailing the accommodations to take place, such as staff training regarding FXS, visual supports and extra time during appointments. A well-designed project that is “FXS-friendly” will ensure that families are met with the expertise and commitment necessary to maximize the opportunity for positive outcomes.

It may be useful to ask family raters (caregivers) what they have heard about the treatment/medication, to yield information about any bias that may be likely in their future assessments. Raters should be given explicit permission to not report improvement if none occurred, to emphasize observed behavior rather than make guesses or inferences or rely overly on third parties, and to remain agnostic to the extent possible about the probability of treatment benefit.

As a patient advocacy organization that has been serving the FXS community of families and professionals for nearly 35 years, the National Fragile X Foundation strongly recommends that parents have a well-defined and meaningful role in providing input regarding clinical trials and new treatment development. There has been a history of unsuccessful trials, parental confusion regarding clinical endpoints, disappointment and, at times, anger over the lack of opportunity for and/or discontinuation of open label extensions among other concerns. Some of these pitfalls can be avoided or, at least, minimized by ensuring that researchers fully understand the worries, concerns, wants, needs, and hopes of parents and other family members. Such input can for example include, among others, recommendations about targets of treatment of importance to families in terms of quality of life, preferred routes of drug administration, advice on the conduct of research procedures within a visit day, feasibility of study designs, and strategies of communication of trials to the community. By providing such an opportunity for family input during protocol development, researchers and, ultimately, those in pharmaceutical industry leadership and decision-making positions, will have access to information that will greatly increase the likelihood of successful trial outcomes and, most importantly, lead to better lives for those with FXS. Development of a family advisory committee as a resource to treatment development programs should meet these needs. 

Understanding the impact of social media on FXS treatment development is also of importance to our field. Because it is not possible to completely monitor Facebook and other types of social media, trial sponsors or investigators should include a stand-alone document on the use of social media and ask parents to sign it in order to participate in the trial. It should state that it is acceptable for participants to post about their experience with the study (e.g., how accommodating the staff is, how to navigate the hospital, tricks they have found successful in helping their family member with FXS participate, and that they are glad to be supporting research). However, the document should also clearly describe what comments are inappropriate because they may bias study reporting such as those about side effects, how the patient is doing, whether they think they are on drug or placebo or how they think other families should rate scales or questionnaires to qualify for the study. 

Many clinical trials have extensively evaluated children and adults with FXS. Assessments might include IQ or equivalent tests, adaptive behavior, language, and testing regarding autism spectrum disorder. Much of this information could be beneficial for families to have for school programming and for additional support services. This testing can be expensive and not readily available to all families. The CTC recommends that relevant findings be shared with parents and guardians and provided to them in a written format that would be readily understood by providers or professionals supporting the child or adult with FXS.

## 7. Multi-Site Trial Planning

Once FXS treatment development has moved to the stage of large-scale Phase II or Phase III investigations, opportunities exist to enhance project executions, fidelity of data gathering, and to facilitate broad human subjects’ recruitment and retention. It is important that within large-scale projects an operations manual that details accommodations to individuals and families with FXS, as the one described in the preceding section, is developed. A well-designed project that is “FXS-friendly” will ensure that families are met with the expertise and commitment necessary to maximize the opportunity for positive outcomes. Rater and investigator training including presentations by FXS thought leaders and family stakeholders will ensure FXS specificity, which will overcome barriers inherent in “off the shelf” central nervous system (CNS) trial approaches grafted into a unique neurodevelopmental disorder setting. 

Such multi-site work should build upon existing FXS centers and clinics with content expertise. Luckily, over a decade of multi-site trial projects in our field has laid a foundation for large-scale trials that has united clinical centers with content expertise with best trial practices and the standard operating procedures of good clinical research. While initial site qualification efforts and trainings should involve FXS content expertise, ongoing support of fidelity of study execution across sites is essential. There are also great opportunities for centralized future potential support of recruitment and retention efforts in large-scale projects given the infrastructure within the FXCRC supported by the National Fragile X Foundation. 

## 8. Summary of Key Recommendations

Much has been learned in the burgeoning FXS treatment development field in the past decade. We have set forth to make many recommendations to enhance the success and stakeholder benefits from future treatment development in our field. From focusing on reproducible early preclinical data to moving towards quantitative markers of pathophysiology as markers of target engagement and change with treatment, clear directions have been defined to broadly support success navigating the chasm between treatment ideas and success in placebo-controlled trials. Along the way we emphasize the importance of engaging the FXS stakeholder community to ensure the meaningfulness of project results and the appropriateness of project procedures from a family and affected individual perspective. Our group remains optimistic at the near-term prospect for impactful treatment development in FXS. We believe that our advocacy and engagement with these processes will work to maximize success and we embrace the opportunity to support the best practices we have defined in this commentary in partnership with treatment developers worldwide. 
